# Standardised inventories of spiders (Arachnida, Araneae) of Macaronesia I: The native forests of the Azores (Pico and Terceira islands)

**DOI:** 10.3897/BDJ.7.e32625

**Published:** 2019-04-16

**Authors:** Jagoba Malumbres-Olarte, Pedro Cardoso, Luís Carlos Fonseca Crespo, Rosalina Gabriel, Fernando Pereira, Rui Carvalho, Carla Rego, Rui Nunes, Maria Teresa Ferreira, Isabel R. Amorim, François Rigal, Paulo A. V. Borges

**Affiliations:** 1 cE3c – Centre for Ecology, Evolution and Environmental Changes / Azorean Biodiversity Group and Universidade dos Açores, Rua Capitão João d’Ávila, São Pedro, 9700-042 , Angra do Heroísmo, Azores, Portugal cE3c – Centre for Ecology, Evolution and Environmental Changes / Azorean Biodiversity Group and Universidade dos Açores, Rua Capitão João d’Ávila, São Pedro, 9700-042 Angra do Heroísmo, Azores Portugal; 2 LIBRe – Laboratory for Integrative Biodiversity Research, Finnish Museum of Natural History, University of Helsinki, Helsinki, Finland LIBRe – Laboratory for Integrative Biodiversity Research, Finnish Museum of Natural History, University of Helsinki Helsinki Finland; 3 IUCN SSC Spider & Scorpion Specialist Group, Helsinki, Finland IUCN SSC Spider & Scorpion Specialist Group Helsinki Finland; 4 Biodiversity Research Institute UB, Department of Evolutionary Biology, Ecology and Environmental Sciences (Athropods), Av. Diagonal 645, E-08028, Barcelona, Spain Biodiversity Research Institute UB, Department of Evolutionary Biology, Ecology and Environmental Sciences (Athropods), Av. Diagonal 645, E-08028 Barcelona Spain; 5 CNRS/ L'Université de Pau et des Pays de l’Adour/ E2S UPPA, Institut Des Sciences Analytiques et de Physico – Chimie pour L'environnement et les Materiaux – MIRA*, UMR5254, 64000, Pau, France CNRS/ L'Université de Pau et des Pays de l’Adour/ E2S UPPA, Institut Des Sciences Analytiques et de Physico – Chimie pour L'environnement et les Materiaux – MIRA*, UMR5254 64000, Pau France; 6 IUCN SSC Mid-Atlantic Islands Specialist Group, Angra do Heroísmo, Azores, Portugal IUCN SSC Mid-Atlantic Islands Specialist Group Angra do Heroísmo, Azores Portugal

**Keywords:** Arthropoda, Araneae, Azores, Terceira, Pico, native forest, exotic species, standardised sampling

## Abstract

**Background:**

The data presented here come from samples collected as part of two recent research projects (NETBIOME - ISLANDBIODIV and FCT - MACDIV) which aimed at understanding the drivers of community assembly in Macaronesian islands. We applied the sampling protocol COBRA (Conservation Oriented Biodiversity Rapid Assessment, Cardoso 2009) in sixteen 50 m x 50 m native forest plots in the Azorean Islands of Pico (6 plots) and Terceira (10 plots) to assess spider diversity. Through this publication, we contribute to the knowledge of the arachnofauna of the Azores and, more specifically, to that of the islands of Pico and Terceira.

**New information:**

The collected samples yielded 8,789 specimens, of which 45% were adults (3,970) belonging to 13 families, 36 species and three morphospecies that have yet to be described. Species of the family Linyphiidae dominated the samples, with 17 species and two morphospecies that have yet to be described (48% of the taxa). Out of the identified (morpho)species, 16 were introduced, 13 Azorean endemic (three of which were undescribed) and seven native (five of them Macaronesian endemics). We report the first record of the introduced species *Haplodrassus signifer* and *Agyneta decora* in Pico Island.

## Introduction

Despite six centuries of disturbance and land use changes, the Azorean islands still contain areas covered by unique native forest (Triantis et al. 2010). This forest accounts for about 5% of the total area of the archipelago and is mainly present on the islands of Flores, Pico and Terceira (Gaspar et al. 2010). From a conservation perspective, the importance of these forests resides in their being home to numerous endemic arthropod species, many of which have been given different status of conservation concern following the IUCN Red List criteria (see for the insects Borges et al. 2017, Borges et al. 2018a). Furthermore, many species – mainly arthropods – await discovery and taxonomic description, despite the high rate of taxonomic descriptions in the last decades (Lobo and Borges 2010). For instance, 24 out of the known 26 endemic spiders have been described since 1989 (Borges and Wunderlich 2008, Borges et al. 2010, Crespo et al. 2013, Crespo et al. 2014).

Indeed, the effective, appropriate and efficient conservation of ecologically valuable areas requires knowing the identity and colonisation status of species (i.e. endemic, native non-endemic or introduced), often provided by large-scale projects such as the EU NETBIOME funded ISLANDBIODIV (*Understanding biodiversity dynamics in tropical and subtropical islands in an aid to science based conservation action*) and the Portuguese FCT funded MACDIV (*Macaronesian Islands as a testing ground to assess biodiversity drivers at multiple scales*). Both ISLANDBIODIV and MACDIV aim to unveil the diversity patterns in vascular plants, springtails, beetles and spiders at local and regional scales in Macaronesian islands (see Emerson et al. 2016, Cicconardi et al. 2017, Borges et al. 2018b). Here we present the information on the species collected in the Azorean forest plots that are part of the ISLANDBIODIV and MACDIV projects.

## Sampling methods

### Study extent

**Figure 1.**
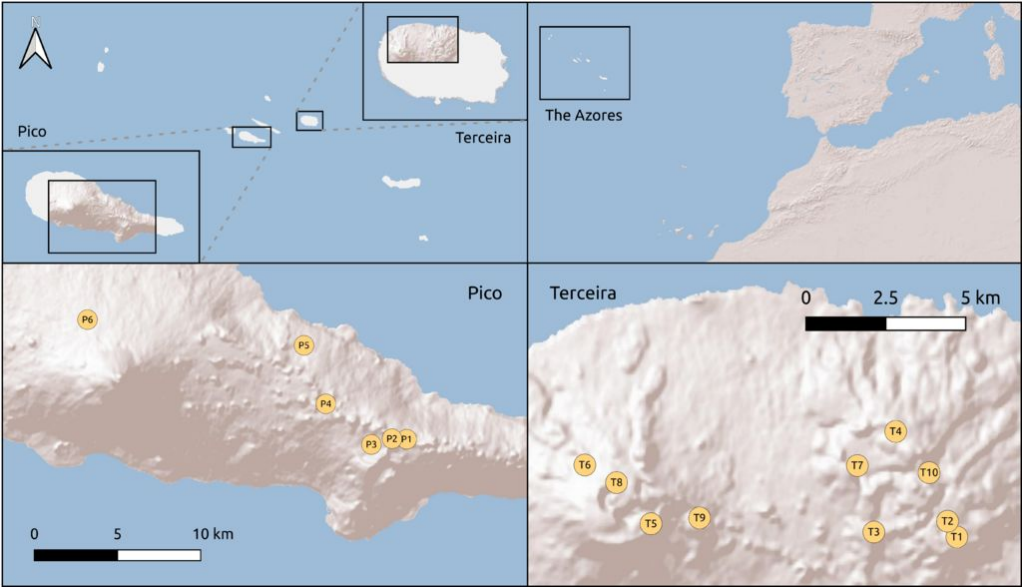
Figure 1. Location of plots in Pico (left) and Terceira (right) islands.

**Figure 2.**
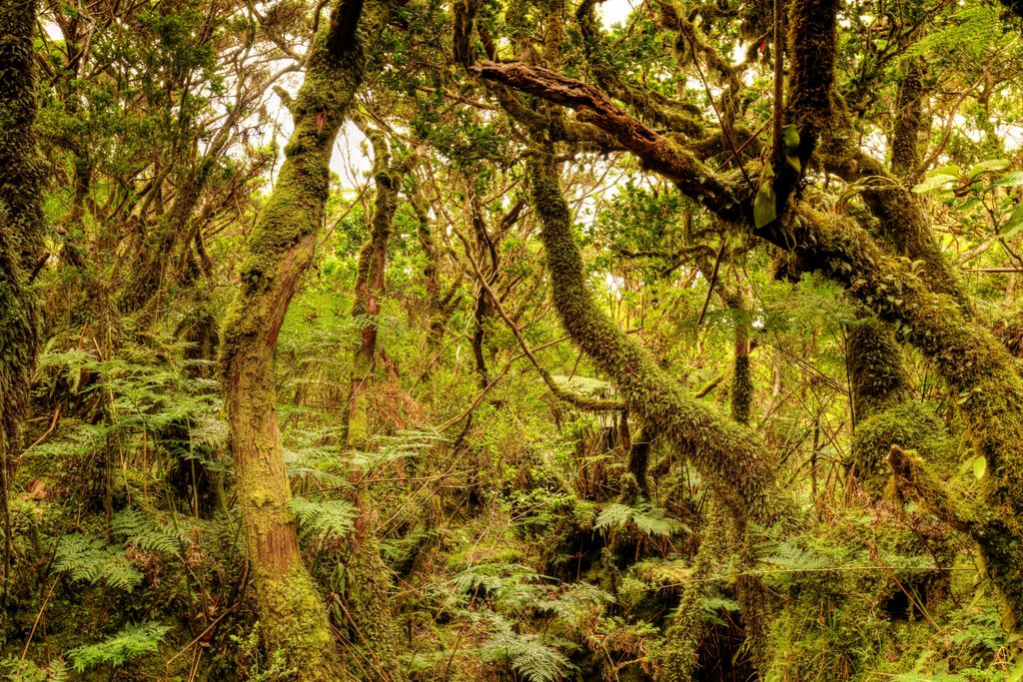
The native forest of Azores: Terra-Brava at Terceira Island - Plot Terceira 1 (Credit: Paulo A. V. Borges).

On each of the Azorean islands of Pico and Terceira, we established six and ten 50 m x 50 m plots along a longitudinal distance of 20 km and 13 km, respectively. In Pico, each plot is located at increasing distances from a first, reference plot (Table 1): 0.1, 1, 5, 10 and 20 km (Fig. 1), covering the three existing forest fragments in order to test distance decay patterns on beta diversity in a log scale within project MACDIV. In Terceira, the 10 plots were randomly distributed in the four main fragments of native forest also to test patterns of alpha and beta diversity (see Cicconardi et al. 2017, Borges et al. 2018b). All plots were located in mid to high elevation native forest dominated by *Juniperus brevifolia*, *Laurus azorica* and Ilex perado subsp. azorica trees (see Borges et al. 2018b for more details on the surveyed habitats) (Fig. 2).

### Sampling description

We sampled all plots using the optimised and standardised COBRA (Conservation Oriented Biodiversity Rapid Assessment) protocol for temperate forests (Cardoso 2009). Different variants of the COBRA protocol for spiders and beetles have already been applied in oceanic islands (Emerson et al. 2016) and for spiders on tropical forests (Malumbres-Olarte et al. 2016, Malumbres-Olarte et al. 2018). Although originally developed and optimised for mainland temperate and Mediterranean habitats, COBRA protocols have been recently proposed as the standard protocols for inventorying and monitoring spiders and beetles in island forest ecosystems (Borges et al. 2018c). The COBRA protocol, when applied to temperate forests, consists of: four night aerial samples (1 hour / sample), two day sweeping samples and two night sweeping samples (1 hour / sample), two day beating samples and two night beating samples (1 hour / sample) and 12 pitfall samples (4 traps / sample). Specifically for islands, we added two sampling methodologies to also cover beetle diversity (Borges et al. 2018c): two diurnal active aerial searching under bark, lichens and bryophytes (ABS) (1 hour / sample) and two diurnal active aerial searching in decaying trunks, dead wood on the ground and under stones (GWS) (1 hour / sample). We collected all samples in July 2016 (Pico, MACDIV) and in June-September 2012 (Terceira, ISLANDBIODIV).

## Geographic coverage

### Description

Pico and Terceira Islands, the Azores, Macaronesia, Portugal

### Coordinates

38.434491 and 38.7617777778 Latitude; -28.4228543692 and -27.1971972222 Longitude.

## Taxonomic coverage

### Taxa included

**Table:** 

Rank	Scientific Name	Common Name
order	Araneae	Spiders

## Temporal coverage

**Single date:** 2012-6-01; 2016-9-26.

## Collection data

### Collection name

Dalberto Teixeira Pombo insect collection at the University of Azores

### Collection identifier

DTP

### Specimen preservation method

All specimens were preserved in 96% ethanol

### Curatorial unit

Dalberto Teixeira Pombo insect collection at the University of Azores (Curator: Paulo A. V. Borges)

## Usage rights

### Use license

Open Data Commons Attribution License

### IP rights notes

CC-BY 4.0

## Data resources

### Data package title

MACDIV_COBRA_Azores_Forest

### Resource link

https://www.gbif.org/dataset/6aa5ac09-2b55-4078-bd2d-ec94fb91850a

### Number of data sets

1

### Data set 1.

#### Data set name

MACDIV_COBRA_Azores_Forest

#### Data format

Darwin Core Archive

#### Number of columns

68

#### Download URL

https://www.gbif.org/dataset/6aa5ac09-2b55-4078-bd2d-ec94fb91850a

#### Data format version

version 1

#### Description

The following data table includes all the records for which a taxonomic identification of the species was possible. The dataset submitted to GBIF is structured as a sample event dataset, with two tables: event (as core) and occurrences. The data in this sampling event resource have been published as a Darwin Core Archive (DwCA), which is a standardised format for sharing biodiversity data as a set of one or more data tables. The core data table contains 423 records (eventID). One extension data table also exists. An extension record supplies extra information about a core record. The number of records in each extension data table is illustrated in the IPT link. This IPT archives the data and thus serves as the data repository. The data and resource metadata are available for downloading in the downloads section. The versions table lists other versions of the resource that have been made publicly available and allows tracking changes made to the resource over time.

In Suppl. material 1, we provide a file with two tables, one with the samping event data and the other with the species abundance data.

**Data set 1.** 

Column label	Column description
Table of Sampling Events	Table with sampling events data (beginning of table)
id	Unique identification code for sampling event data
eventID	Identifier of the events, unique for the dataset
samplingProtocol	The sampling protocol used to capture the species
sampleSizeValue	The numeric amount of time spent in each sampling
sampleSizeUnit	The unit of the sample size value
samplingEffort	The amount of time of each sampling
eventDate	Date or date range the record was collected
eventTime	Time of the day the record was collected
startDayOfYear	The earliest ordinal day of the year on which the event occurred
endDayOfYear	The latest ordinal day of the year on which the event occurred
year	Year of the event
month	Month of the event
day	Day of the event
habitat	The surveyed habitat
fieldNumber	The code given to each sample
locationID	Identifier of the location
islandGroup	Name of archipelago
island	Name of the island
country	Country of the sampling site
countryCode	ISO code of the country of the sampling site
stateProvince	Name of the region of the sampling site
municipality	Name of the municipality
locality	Name of the locality
minimumElevationInMetres	Minimum elevation in metres
maximumElevationInMetres	Maximum elevation in metres
locationRemarks	Details on the locality site
verbatimCoordinates	The Verbatim coordinates
decimalLatitude	Approximate centre point decimal latitude of the field site in GPS coordinates
decimalLongitude	Approximate centre point decimal longitude of the field site in GPS coordinates
geodeticDatum	The reference point for the various coordinate systems used in mapping the earth
coordinateUncertaintyInMetres	Uncertainty of the coordinates
coordinatePrecision	Precision of the coordinates
georeferenceSources	A list (concatenated and separated) of maps, gazetteers or other resources used to georeference the Location, described specifically enough to allow anyone in the future to use the same resources.
Table of Species Abundances	Table with species abundance data (beginning of new table)
id	Unique identification code for species abundance data
type	Type of the record, as defined by the Public Core standard
licence	Reference to the licence under which the record is published
institutionID	The identity of the institution publishing the data
collectionID	The identity of the collection publishing the data
institutionCode	The code of the institution publishing the data
collectionCode	The code of the collection where the specimens are conserved
datasetName	Name of the dataset
basisOfRecord	The nature of the data record
dynamicProperties	The name of the scientific project funding the sampling
occurrenceID	Identifier of the record, coded as a global unique identifier
catalogNumber	Record number of the specimen in the collection
recordedBy	Name of the person who performed the sampling of the specimens
individualCount	Total number of individuals captured
organismQuantity	Total number of individuals captured, as numeric
organismQuantityType	The unit of the identification of the organisms
sex	The sex and quantity of the individuals captured
lifeStage	The life stage of the organisms captured
establishmentMeans	The process of establishment of the species in the location, using a controlled vocabulary: 'native non-endemic', 'introduced', 'endemic'
occurrenceStatus	Information about the presence/absence of the species
eventID	A unique identifier of an occurrence
identifiedBy	Name of the person who made the identification
dateIdentified	Date on which the record was identified
scientificName	Complete scientific name including author and year
kingdom	Kingdom name
phylum	Phylum name
class	Class name
order	Order name
family	Family name
genus	Genus name
specificEpithet	Specific epithet
taxonRank	Lowest taxonomic rank of the record
scientificNameAuthorship	Name of the author of the lowest taxon rank included in the record

## Additional information

### Results

We collected 3,930 adult specimens (45% of all specimens), which we identified to 36 species and three morphospecies, belonging to 13 families (Tables 2, 3). Of the 39 taxa, 19 belonged to the family Linyphiidae (17 species and two morphospecies) and six species to Theridiidae. The remaining families were represented by one or two taxa. The most widespread species were *Gibbaranea occidentalis* Wunderlich, 1989 (endemic), *Lathys dentichelis* (Simon, 1883) (native), *Acorigone acoreensis* (Wunderlich, 1992) (endemic), *Tenuiphantes miguelensis* (Wunderlich, 1992) (Macaronesian), *Macaroeris cata* (Blackwall, 1867) (native) and *Sancus acoreensis* (Wunderlich, 1992) (endemic), which were present in all plots of both islands. Three additional species were present in 15 out of the 16 plots: *Canariphantes acoreensis*, *Microlinyphia johnsoni* and *Savigniorrhipis acoreensis*. Plots had between 15-23 (morpho)species of spiders, with the Terceiran Plot 8 having the maximum of 23 (morpho)species, followed by Plot 3 of Terceira (21 species).

**Figure 3.**
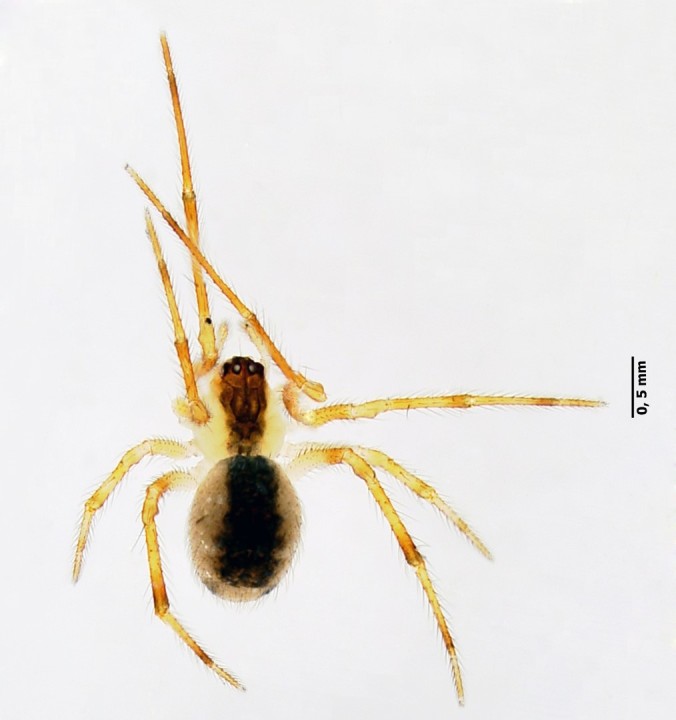
*Rugathodes acoreensis* Wunderlich, 1992 (Credit: Enésima Mendonça, Azorean Biodiversity Portal).

**Figure 4.**
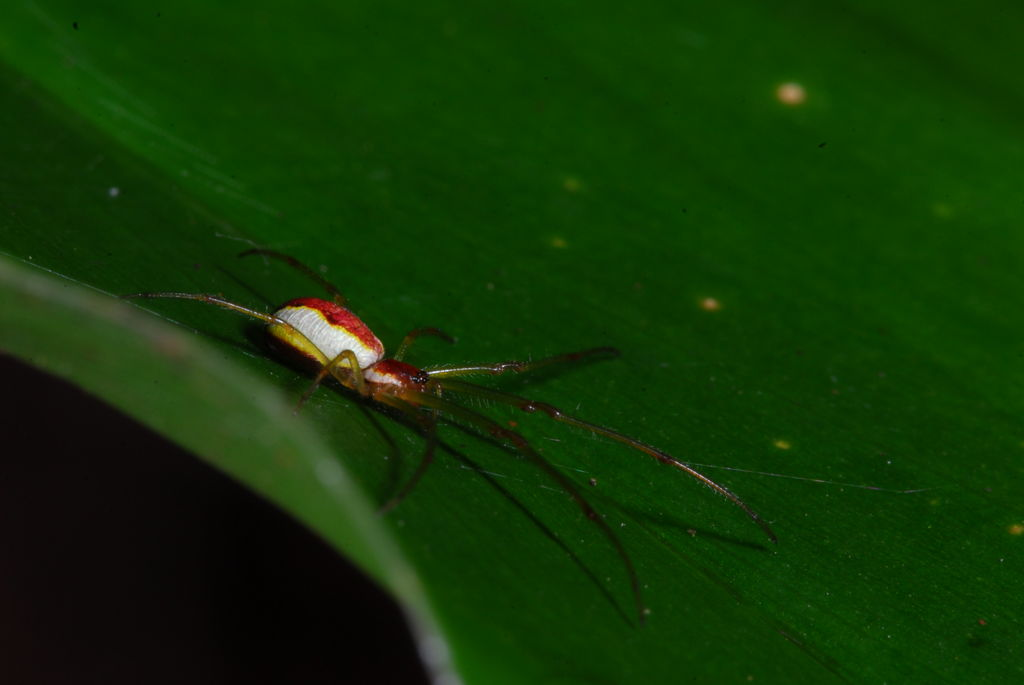
*Sancus acoreensis* (Wunderlich, 1992) (Credit: Pedro Cardoso).

**Figure 5.**
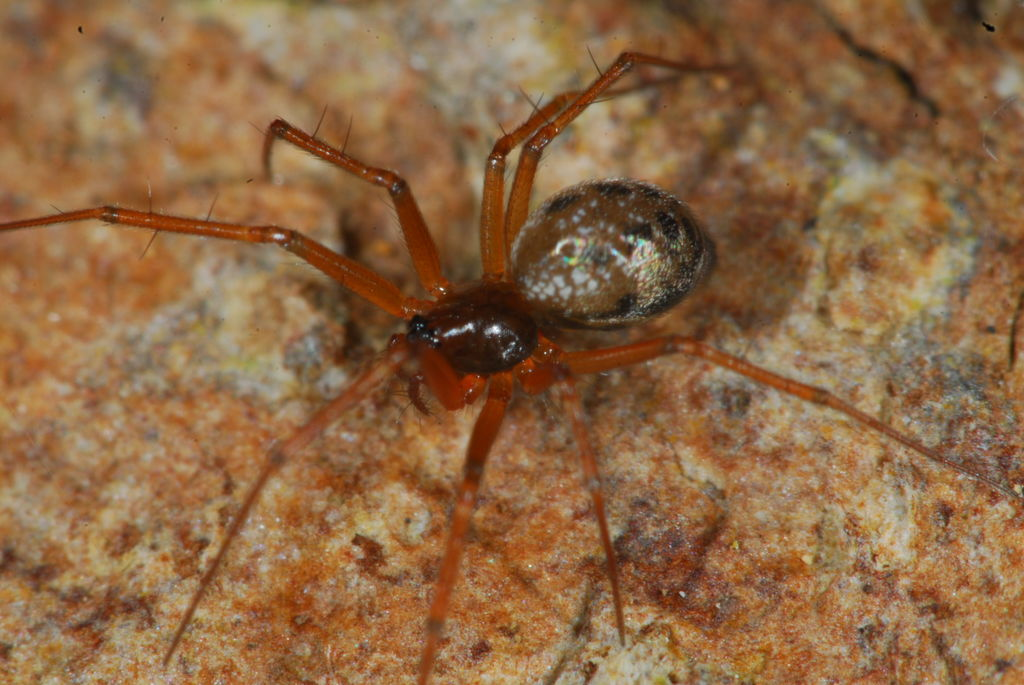
*Tenuiphantes miguelensis* (Wunderlich, 1992) (Credit: Pedro Cardoso).

**Figure 6.**
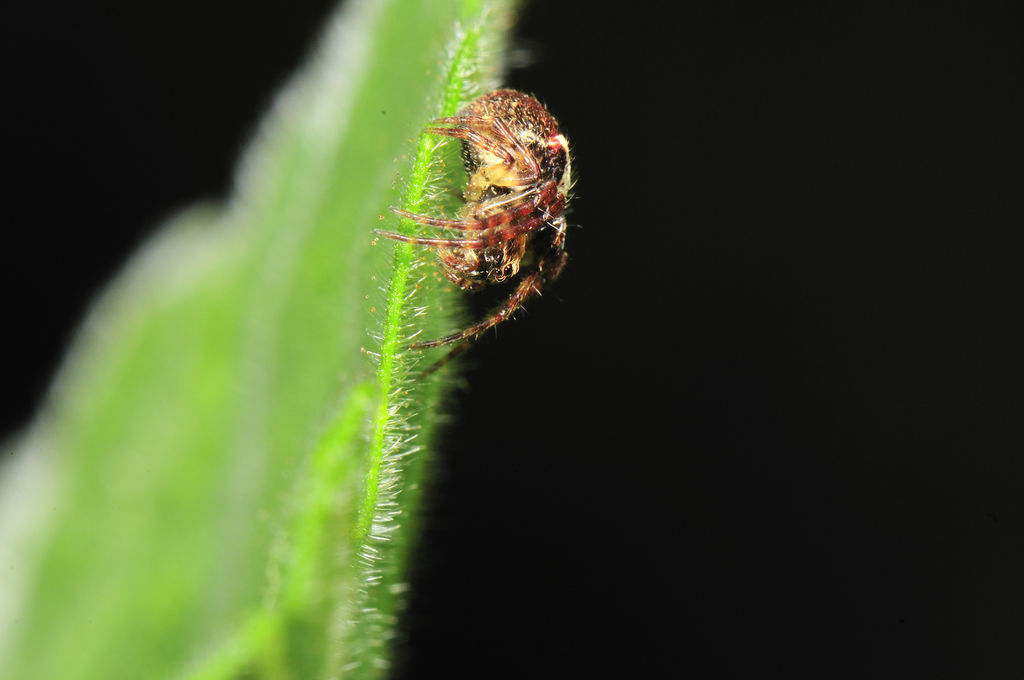
*Gibbaranea occidentalis* Wunderlich, 1989 (Credit: Paulo A. V. Borges).

Four species accounted for 54% of all specimens: *Rugathodes acoreensis* (753 specimens) (Fig. 3), *Sancus acoreensis* (506) (Fig. 4), *Tenuiphantes miguelensis* (479) (Fig. 5) and *Gibbaranea occidentalis* (392) (Fig. 6). These species occur in different forest micro-habitats: the orb-weaver, *G. occidentalis*, occurs in both canopy and intermediate understorey; the theridiid, *R. acoreensis*, is mostly a canopy species; the tetragnathid, *S. acoreensis*, is usually associated with shrubs; and *T. miguelensis* is a forest ground linyphiid, building its sheet-webs between small holes in the ground or small crevices in volcanic rocks.

Of the 39 collected species, 34 have been recorded previously in both Pico and Terceira islands (Borges et al. 2010). In total, we recorded 13 endemic species, 16 introduced, five Macaronesian, two native and three of unknown biogeographic category but possibly also endemic (which will be subject to a molecular and morphological integrative taxonomic description). Two of the 16 introduced species (*Haplodrassus signifer* and *Agyneta decora*) were recorded in Pico for the first time (cf. Borges et al. 2010).

## Supplementary Material

Supplementary material 1 MACDIV_ISLANDBIOD_Spiders_Azores_Base_GBIF Data type: Species abundances and sampling events
Brief description: In this contribution, we present detailed data on the distribution and abundance of spider species found in Azores forest plots (six in Pico and 10 in Terceira).
File: oo_268313.xls Malumbres-Olarte, J et al.

## Figures and Tables

**Table 1.** Coordinates of sampling plots.

Plot	Longitude	Latitude
Pico 1	-28.2017136846	38.437588
Pico 2	-28.2117989478	38.437737
Pico 3	-28.2259599451	38.434491
Pico 4	-28.257662125	38.4561062785
Pico 5	-28.2733451278	38.4876669302
Pico 6	-28.4228543692	38.4998686917
Terceira 1	-27.1971972222	38.7320583333
Terceira 2	-27.2005772537	38.7364977463
Terceira 3	-27.2271119278	38.7334147054
Terceira 4	-27.2193222222	38.7617777778
Terceira 5	-27.3074033132	38.7355657746
Terceira 6	-27.3313027778	38.7520777778
Terceira 7	-27.233098	38.75214
Terceira 8	-27.3198583333	38.7471444444
Terceira 9	-27.2899410553	38.7372469297
Terceira 10	-27.2072466047	38.7501938461

**Table 2.** Abundance, biogeographic category and previous records of (morpho)species in each of the plots on Pico island. Abbreviations: Biogeographic category (Biog. cat): Endemic (END); Introduced (INT); Macaronesian (MAC); Native (NAT); Unknown (UK). Previous records (Prev. Rec.): Pico (P), Terceira (T), unrecorded (UR).

**Family**	**Species**	**Biog.Cat.**	**Prev.Rec.**	**Pico 1**	**Pico 2**	**Pico 3**	**Pico 4**	**Pico 5**	**Pico 6**
Araneidae	*Gibbaranea occidentalis* Wunderlich, 1989	END	P, T	12	4	4	7	14	23
Clubionidae	*Cheiracanthium erraticum* (Walckenaer, 1802)	INT	P, T	0	0	1	0	0	1
Dictynidae	*Lathys dentichelis* (Simon, 1883)	MAC	P, T	23	31	10	6	7	1
Dictynidae	*Nigma puella* (Simon, 1870)	INT	P, T	0	0	0	0	6	0
Dysderidae	*Dysdera crocata* C. L. Koch, 1838	INT	P, T	0	0	0	1	3	0
Gnaphosidae	*Haplodrassus signifer* (C. L. Koch, 1839)	INT	T	0	1	0	0	0	0
Linyphiidae	Linyphiidae morphospecies 1220	UK	UR	0	0	0	0	0	0
Linyphiidae	Linyphiidae morphospecies 1265	UK	UR	3	0	0	0	0	0
Linyphiidae	*Acorigone acoreensis* (Wunderlich, 1992)	END	P, T	6	5	7	1	1	1
Linyphiidae	*Agyneta decora* (O. P.-Cambridge, 1871)	INT	T	0	0	0	0	0	0
Linyphiidae	*Canariphantes acoreensis* (Wunderlich, 1992)	END	P, T	2	12	0	7	6	6
Linyphiidae	*Erigone atra* Blackwall, 1833	INT	P, T	0	1	1	1	0	2
Linyphiidae	*Erigone autumnalis* Emerton, 1882	INT	P, T	0	0	2	0	0	0
Linyphiidae	*Erigone dentipalpis* (Wider, 1834)	INT	P, T	0	0	2	0	0	0
Linyphiidae	*Mermessus bryantae* (Ivie & Barrows, 1935)	INT	P, T	0	0	0	0	0	0
Linyphiidae	*Microlinyphia johnsoni* (Blackwall, 1859)	MAC	P, T	5	5	3	35	0	7
Linyphiidae	*Minicia floresensis* Wunderlich, 1992	END	P, T	18	4	7	0	0	0
Linyphiidae	*Oedothorax fuscus* (Blackwall, 1834)	INT	P, T	0	1	0	4	0	0
Linyphiidae	*Palliduphantes schmitzi* (Kulczynski, 1899)	MAC	P, T	1	0	0	2	0	9
Linyphiidae	*Porrhomma borgesi* Wunderlich, 2008	END	P, T	0	0	0	0	0	0
Linyphiidae	*Prinerigone vagans* (Audouin, 1826)	INT	P, T	0	0	0	0	0	0
Linyphiidae	*Savigniorrhipis acoreensis* Wunderlich, 1992	END	P, T	15	31	10	15	0	13
Linyphiidae	*Tenuiphantes miguelensis* (Wunderlich, 1992)	MAC	P, T	4	5	10	102	107	73
Linyphiidae	*Tenuiphantes tenuis* (Blackwall, 1852)	INT	P, T	2	1	8	4	3	5
Linyphiidae	*Walckenaeria grandis* (Wunderlich, 1992)	END	P, T	13	3	0	0	0	0
Lycosidae	*Pardosa acorensis* Simon, 1883	END	P, T	28	8	24	1	0	0
Mimetidae	*Ero furcata* (Villers, 1789)	INT	P, T	0	0	0	0	1	0
Pisauridae	*Pisaura acoreensis* Wunderlich, 1992	END	P, T	1	1	0	0	0	0
Salticidae	*Macaroeris cata* (Blackwall, 1867)	NAT	P, T	12	8	13	1	5	3
Salticidae	*Neon acoreensis* Wunderlich, 2008	END	P, T	0	0	0	0	4	0
Tetragnathidae	*Metellina* sp. 133	UK	T	0	0	0	0	0	0
Tetragnathidae	*Metellina merianae* (Scopoli, 1763)	INT	P, T	4	4	2	17	51	8
Tetragnathidae	*Sancus acoreensis* (Wunderlich, 1992)	END	P, T	35	38	19	14	11	50
Theridiidae	*Lasaeola oceanica* Simon, 1883	END	P, T	0	0	12	0	8	2
Theridiidae	*Neottiura bimaculata* (Linnaeus, 1767)	INT	P, T	0	0	0	0	2	0
Theridiidae	*Rugathodes acoreensis* Wunderlich, 1992	END	P, T	39	61	69	0	0	19
Theridiidae	*Steatoda nobilis* (Thorell, 1875)	MAC	P, T	0	0	0	0	4	0
Theridiidae	*Theridion melanurum* Hahn, 1831	INT	P,	0	0	0	0	19	1
Thomisidae	*Xysticus cor* Canestrini, 1873	NAT	P, T	3	6	10	1	0	2
Species richness				19	20	19	17	17	18

**Table 3.** Abundance, biogeographic category and previous records of (morpho)species in each of the plots on Terceira island.

**Species**	**Plot 1**	**Plot 2**	**Plot 3**	**Plot 4**	**Plot 5**	**Plot 6**	**Plot 7**	**Plot 8**	**Plot 9**	**Plot 10**	**Total**
*Gibbaranea occidentalis* Wunderlich, 1989	33	25	49	30	4	32	39	22	42	52	392
*Cheiracanthium erraticum* (Walckenaer, 1802)	0	1	0	3	3	10	0	3	0	1	23
*Lathys dentichelis* (Simon, 1883)	17	12	19	27	15	8	11	13	24	27	251
*Nigma puella* (Simon, 1870)	0	0	0	0	0	0	0	0	0	0	6
*Dysdera crocata* C. L. Koch, 1838	1	0	4	0	0	0	6	0	2	0	17
*Haplodrassus signifer* (C. L. Koch, 1839)	0	0	0	0	0	0	0	0	0	0	1
Linyphiidae morphospecies 1220	0	0	0	0	2	0	0	0	0	0	2
Linyphiidae morphospecies 1265	0	0	0	0	0	0	0	0	0	0	3
*Acorigone acoreensis* (Wunderlich, 1992)	6	6	2	6	12	3	9	12	3	2	82
*Agyneta decora* (O. P.-Cambridge, 1871)	0	0	1	0	3	0	0	3	0	0	7
*Canariphantes acoreensis* (Wunderlich, 1992)	9	13	12	10	22	3	6	8	4	1	121
*Erigone atra* Blackwall, 1833	0	0	1	0	0	1	0	0	0	0	7
*Erigone autumnalis* Emerton, 1882	0	0	0	0	0	0	0	2	0	0	4
*Erigone dentipalpis* (Wider, 1834)	0	0	0	0	0	0	0	0	0	0	2
*Mermessus bryantae* (Ivie & Barrows, 1935)	0	0	0	0	0	0	0	1	0	0	1
*Microlinyphia johnsoni* (Blackwall, 1859)	4	30	2	8	11	13	9	5	6	5	148
*Minicia floresensis* Wunderlich, 1992	0	1	0	1	0	0	1	4	0	5	41
*Oedothorax fuscus* (Blackwall, 1834)	0	0	1	0	0	0	0	1	0	0	7
*Palliduphantes schmitzi* (Kulczynski, 1899)	0	0	0	0	0	2	0	0	0	0	14
*Porrhomma borgesi* Wunderlich, 2008	0	1	2	2	1	0	0	4	1	1	12
*Prinerigone vagans* (Audouin, 1826)	0	1	0	0	0	0	0	0	0	1	2
*Savigniorrhipis acoreensis* Wunderlich, 1992	26	63	23	22	11	56	16	16	15	35	367
*Tenuiphantes miguelensis* (Wunderlich, 1992)	18	13	11	18	4	36	5	39	10	24	479
*Tenuiphantes tenuis* (Blackwall, 1852)	0	0	0	1	0	1	1	1	1	0	28
*Walckenaeria grandis* (Wunderlich, 1992)	0	0	0	1	10	0	0	28	0	1	56
*Pardosa acorensis* Simon, 1883	0	4	1	1	22	0	1	23	0	0	113
*Ero furcata* (Villers, 1789)	4	2	5	1	0	0	3	0	3	3	22
*Pisaura acoreensis* Wunderlich, 1992	2	3	3	1	0	0	1	2	4	9	27
*Macaroeris cata* (Blackwall, 1867)	6	9	1	15	2	16	5	3	14	10	123
*Neon acoreensis* Wunderlich, 2008	0	0	0	0	0	0	1	0	0	0	5
*Metellina* sp. 133	6	11	33	4	8	4	14	2	2	1	85
*Metellina merianae* (Scopoli, 1763)	0	0	0	0	0	0	0	0	0	0	86
*Sancus acoreensis* (Wunderlich, 1992)	37	26	37	26	34	32	56	34	16	41	506
*Lasaeola oceanica* Simon, 1883	4	2	8	11	3	3	3	0	14	9	79
*Neottiura bimaculata* (Linnaeus, 1767)	0	0	0	0	0	0	0	0	0	0	2
*Rugathodes acoreensis* Wunderlich, 1992	31	33	78	40	123	0	48	54	35	123	753
*Steatoda nobilis* (Thorell, 1875)	0	0	0	0	0	0	0	0	0	0	4
*Theridion melanurum* Hahn, 1831	0	0	0	0	0	0	0	0	0	0	20
*Xysticus cor* Canestrini, 1873	0	1	3	0	0	0	0	4	0	2	32
Species richness	15	20	21	20	18	15	19	23	17	20	39
